# A feasibility study of a new instrument for detection of autism signs in preschool settings. Psychometric properties of the joint attention observation schedule preschool (JA-OBS preschool)

**DOI:** 10.3389/frcha.2022.1061451

**Published:** 2023-01-18

**Authors:** Petra Linnsand, Jonas Hermansson, Christopher Gillberg, Gudrun Nygren

**Affiliations:** ^1^ Gillberg Neuropsychiatry Centre, Institute of Neuroscience and Physiology, University of Gothenburg, Gothenburg, Sweden; ^2^ Child and Adolescent Specialist Centre, Angered Hospital, SV Hospital Group, Gothenburg, Sweden; ^3^ Research department, Angered hospital, SV Hospital Group, Gothenburg, Sweden

**Keywords:** autism, early detection, preschool, psychometric properties, multiethnic low resource setting

## Abstract

**Background:**

Identifying signs of autism is essential for enabling timely diagnosis and intervention. Children from multiethnic and socioeconomic disadvantaged settings are typically diagnosed with autism later than their general peers. This feasibility study explored the psychometric properties of a new instrument, Joint Attention Observation Schedule Preschool (JA-OBS Preschool), in children with suspected autism.

**Methods:**

Data were collected from a prospective longitudinal study of 46 children aged 2–4 years who lived in a multiethnic, low resource area. The children had been referred from the Child Health Centre to a multiprofessional team for a neuropsychiatric assessment. In the diagnostic process, the instrument JA-OBS Preschool for observation of a child's capability of engaging in joint attention was included. Descriptive statistics and Cronbach's alfa were used to analyze the psychometric properties, including internal consistency reliability and inter-rater consistency.

**Results:**

All 46 children with suspected autism screened positive on the JA-OBS Preschool. The internal consistency reliability of the instrument was calculated at 0.8 (Cronbach's alfa). Percent agreement between two examiners in JA-OBS Preschool ranged from 77% to 100%.

**Conclusion:**

JA-OBS Preschool was found to be a promising instrument for identifying signs of autism in children in this setting. However, further research is needed to determine the psychometric properties of the JA-OBS Preschool in a general child population as well as in a younger age group.

## 1. Introduction

Autism Spectrum Disorder is a lifelong neurodevelopmental disorder that emerges in early childhood and characterized by impairments in social interaction and communication, as well as in repetitive pattern of behaviours and interests and/or unusual sensory reactions. Autism is associated with significant impairment in everyday functioning (1) and is highly correlated with co-existing conditions, such as language disorders and intellectual developmental disorder (2, 3). Approximately 1%–2% of preschool children in a general population receive an autism diagnosis (4, 5). Studies have indicated a higher prevalence of autism among children of immigrants (6–9). The etiology of autism is complex involving genetic, epigenetic and environmental factors (10, 11). Genetics play a key role in the etiology (12–15), but different factors influence each other (10). Still, the exact etiology remains unknown for the majority of cases. However, early detection of autism is critical to facilitate early intervention to improve developmental outcomes and prevent secondary behavioural problems (16–21). Early autism intervention can also enhance how families adapt and reduce parental stress (22) and societal costs (23).

Early symptoms of autism may emerge during the first years of life, and are manifested as abnormalities in social communication and repetitive behaviours. Impairments in social communication include reduced social engagement, deficits in developing relationship, reduced orienting to name, delayed language development without compensatory pointing or gesturing, and reduced joint attention behaviour (19, 24–27). Joint attention behaviours refer to the ability to coordinate visual attention between two persons and an external object in order to share attention (28, 29). In typically developing children, joint attention emerges in early infancy and continues to develop into more coordinated and complex behaviour between 8 and 18 months of age (24, 28). Children with autism have particular difficulty engaging in joint attention activities, including both initiating joint attention and responding to joint attention attempts of others (25, 28). Children not demonstrating these abilities after 15 months of age should be assessed for autism (30), and even earlier in infants at high familial risk for autism (31, 32).

Several studies have demonstrated that a reliable autism diagnosis can be made from 14 months of age (18, 30, 33–35). In clinical recommendations, screening for autism is recommended at 18- and 24- or 30-month well-child visits (19, 30, 36). A review of early detection procedures for autism in primary care and other community settings have found that routine screening increased overall referral rates (37). Furthermore, screening measures have been shown to be more effective in identifying children with autism than clinical judgment or caregiver concerns alone (38), and together with diagnostic tools for autism, screening has allowed diagnoses at younger ages (39).

Several screening instruments are available for the detection of autism among young children. The most widely used and studied screening tool is the Modified Checklist for Autism in Toddlers (M-CHAT/M-CHAT-R/F) (40, 41). The M-CHAT-R/F is a parent-rated scale, and a positive finding leads to a follow-up interview of health care professionals. The M-CHAT-R/F was found to be an effective tool for screening toddlers, decreasing the age of diagnosis by two years compared to the U.S. national median age of diagnosis (42, 43). Some screening methods use a combination of the parental report and clinical judgement, e.g., the original Checklist for Autism in Toddlers (CHAT) (44–46). Some later developed observation-based screening instruments, all focusing on the early social communication, are the Screening Tool for Autism in Two-Year-Olds (STAT) (47, 48), the Autism Detection in Early Childhood (ADEC) (49), and the Social Attention and Communication Surveillance (SACS) (50). The 5-item instrument Joint Attention Observation Schedule (JA-OBS) for clinical judgement in Child Health Care (CHC) was developed in our group for use in combination with the parent-rated M-CHAT-R (45). In a study by Nygren et al. (45), the positive predictive value for the combination of screening tools was high (89.6%).

Several studies have highlighted that preschool staff can effectively detect early symptoms of autism and other atypical features in early development (51–55), also in low-income and multiethnic settings (56, 57). Day-care workers often have education in child development, as well as experience in working with children with diverse disabilities. In a Swedish study, Nilsson Jobs et al. (58) showed that compared to parents, preschool professionals were able to identify children with autism, and their ratings were more in accordance with clinician-rated symptoms. Considering that developmental disabilities affect at least 8 to 10% of young children (59, 60), preschool may be an essential setting for early detection of autism and other developmental disabilities. In addition, in many countries, many children spend much of their time in a community childcare setting. In Sweden, 90.6% of children aged two years attend preschool (61).

Even though autism can be detected in the first two years of life, the recommendations for screening, and access to effective methods for early identification have not yet become the norm in many countries (19, 62–65). Delayed diagnosis of autism may be even more prevalent in areas with a high prevalence of people with low-income and/or multiethnic backgrounds (8, 44, 66–70).

Therefore, it is important to develop new methods for early detection of autism, which can complement the screening methods already used in health care, this need is emphasized in areas with a high prevalence of families with multiethnic background. The present study is a feasibility study of a new instrument for detection of autism signs in a preschool setting. The aim was to explore the psychometric properties of this new instrument, named Joint Attention Observation Schedule Preschool (JA-OBS Preschool) in children with suspected autism.

## 2. Materials and methods

### 2.1. The study setting

The study area consists of one district of Gothenburg, Sweden, with ∼13,200 inhabitants of whom ∼1,350 were children aged 0–5 years. The district is one of the most multiethnic and socioeconomically disadvantaged areas in Gothenburg, where around 90% of the population has a foreign background. The area has a high prevalence of ill health, unemployment, and low income (71). In an earlier study from this area, we have reported a high prevalence of autism, 3.66%, in preschool children (7). For more details regarding the study area, see Linnsand et al. (7).

### 2.2. The healthcare setting

In Sweden, healthcare is mainly tax-funded and is primarily regulated by the Health and Medical Service Act (SFS 2017:30) (72). Health care is free for all children, CHC has a developmental surveillance program for children between 0 and 6 years and reach about 99% of all children (73).

### 2.3. The preschool setting

In Sweden, preschool is not compulsory, but approximately 522,000 children (85.4%) aged 1–5 years old attend preschool. Of children aged 1–3 years, approximately 286,000 (78.6%) attend preschool. Of the children aged 1–3 years old with foreign backgrounds, 75.5% attend preschool (61). Preschool education represents the first step in the Swedish educational system and is included in the Swedish Education Act (74). The preschool curriculum contains the fundamental values and tasks of the preschool and goals and guidelines (75). In Sweden, nearly all children with special educational needs are enrolled in regular preschools. Professionals in Swedish preschool mainly include preschool teachers (university degree in Preschool Education) and preschool care workers (upper secondary education).

### 2.4. Participants

This study is based on 46 children (9 girls, 37 boys) born between 2010 and 2016 and diagnosed with DSM-5 autism (American Psychiatric Association 2013). The average age for the autism diagnosis was 38 months (range = 22–59 months, standard deviation = 9). All children, but one, lived in the same socioeconomic disadvantaged area in Gothenburg. Most of their parents had a non-Swedish background (93.5%).

### 2.5. Procedure

Data were collected from an ongoing prospective longitudinal study of young children diagnosed with autism. Medical records from the CHC and the comprehensive neuropsychiatric assessment were reviewed by two of the authors (PL and GN) independently. Extracted data included results from the autism screening at CHC, demographics, and the diagnostic assessment results. For more details regarding data collection, see Linnsand et al. (7).

#### 2.5.1. The screening procedure at the child health centre

**Figure 1:**
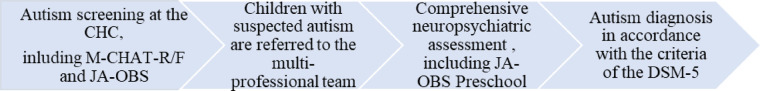
From the autism screening at the CHC to the comprehensive neuropsychiatric assessment.

In the city of Gothenburg, all 30-month-old children are invited to autism screening at the CHC (Figure 1). The screening includes a combination of instruments: the parental questionnaire M-CHAT-R/F and the clinical observation of the child, the JA-OBS (Table 1, item 1–5) (45). The methods are also used if there is suspicion of autism before the routine screening. Children with suspected autism or other neurodevelopmental disorders at the CHC are referred to a multiprofessional team for further assessment.

**Table 1:** JA-OBS (item 1–5) (45) and JA-OBS preschool (item 1–7).

Does the child…
1. React to own name (turns to person addressing the child)? 2. Try to establish eye contact with you? 3. Gaze at something that you point to further away in the room? 4. Use his/her index-finger to point at something (e.g., in a book)? 5. Interact with you or parent in pretend play (e.g., during feeding a doll, or putting the doll to bed; does the child use eye contact to monitor that you are watching)? 6. Show interest in other children? 7. Take some initiative to contact others for play, to show something, to tell something, not just because others are looking for contact?

#### 2.5.2. The performance of the Ja-OBS preschool

The neuropsychiatric assessment included an observation of the child in the preschool environment (Figure 1). A special education teacher or a psychologist from the assessment team observed the child in free play and group activities. Also, an interview with the preschool teachers concerning the child's abilities and behaviour in different everyday activities was performed. During the observation, the examiner performed the JA-OBS Preschool (Table 1, item 1–7). The examiner interacted with the child and presented the tasks to him/her, for example, introducing the play, e.g., feeding a doll. Three children in the study group had no placement in any preschool. In these cases, the psychologist observed the child in the home environment, including the JA-OBS (Table 1, item 1–5).

For 13 children, one of two special education teachers from the preschool also performed the JA-OBS Preschool. The preschool special education teachers had received special training in how to perform the observation. The education focused on the typical development of communication and interaction, including joint attention behaviours, and early signs of autism, as well as the practical use of the instrument. The training also included case descriptions, where the different items were discussed.

The preschool special education teachers (the preschool setting) and the psychologist from the assessment team (the health care setting) did not observe the child simultaneously but during the same two-week period. The examiners were not aware of the other's ratings.

#### 2.5.3. The diagnostic procedure at the multiprofessional team

A comprehensive neuropsychiatric assessment was performed for the 46 children, which included a physical developmental examination, a parental interview regarding the child's developmental and medical history, current clinical symptoms and social situation, and assessment of the child's intellectual level [The Wechsler Preschool and Primary Scale of Intelligence, Fourth Edition (WPPSI-IV) (76) for the children over 2:6 years, and for younger children Merrill-Palmer—Revised Scales of Development (MP-R) (77)]. The Autism Diagnostic Observation Schedule (ADOS) (78) or the Autism Diagnostic Observation Schedule Second Edition (ADOS-2) (79) were included. The Vineland Adaptive Behavior Scales Second Edition (VABS-II) (80) was performed with the parents. In addition, an observation of the child in the preschool environment, including JA-OBS Preschool, was also included in the assessment. The autism diagnosis was based on all obtained information from the assessments and in accordance with the criteria of the DSM-5 (299.00, Autism Spectrum Disorder) (1). The multiprofessional team and the assessment are further described in Linnsand et al. (7).

### 2.6. Measures

#### 2.6.1. Joint attention observation schedule

The JA-OBS is a five-item observation of a child's capability of engaging in joint attention activities (initiation of joint attention and response to joint attention). The observation also includes other social communicative behaviours, i.e., the child's response to own name and the ability to interact in pretend play (Table 1, item 1–5). Nygren et al. (45) developed the JA-OBS based on results received in studies of early autism symptoms related to reduced joint attention behaviours. The screen positivity for autism on the JA-OBS occurred when the examiners did not observe the target behaviours on two or more of the items.

#### 2.6.2. Joint attention observation schedule preschool

The JA-OBS Preschool is a further development of the JA-OBS for use in preschool (developed by the author GN). The JA-OBS Preschool contains the first five-item from the JA-OBS (Table 1, item 1–5) plus two additional items (Table 1, item 6–7). The additional items focus on aspects of social communication that can be observed in a preschool setting. Based on previous research of JA-OBS and the clinical experience of the JA-OBS Preschool, a positive screening result for autism was defined as failure on two or more of the seven items (45).

### 2.7. Statistical analysis

*Internal consistency reliability* was calculated using Cronbach's alfa. *Inter-rater consistency* was calculated as percent agreement. The calculations were performed using SPSS version 27 (81).

## 3. Results

### 3.1. Sample characteristics

The study group consisted of 46 children (9 girls, 37 boys) diagnosed with autism in accordance with DSM-5 (1). Regarding autism severity level, 25 children (54.3%) had autism level 1, and 21 children (45.7%) had autism level 2. Six children (13%) had average intellectual functioning (Intelligence Quotient (IQ)/Developmental Quotient (DQ) ≥85), 12 children (26.1%) had borderline intellectual functioning (IQ/DQ 70–84), and 28 children (60.9%) intellectual disability (IQ/DQ <70).

### 3.2. Results of Ja-OBS and Ja-OBS preschool

Of the 46 children, 38 had JA-OBS completed from CHC, and 41 children had JA-OBS Preschool completed for them. On the JA-OBS, 32 children screened positive for autism (failure on ≥2 of the five items), whereas all 41 children screened positive on JA-OBS Preschool (failure on ≥2 of the seven items) (Table 2). Of the children, 88.2% screened positive on both JA-OBS and JA-OBS Preschool.

**Table 2:** Results of JA-OBS and JA-OBS preschool, number (*n*) and percentage (%), *n* = 46.

	Screen positive, *n* (%)	Screen negative, *n* (%)	Data missing, *n* (%)
JA-OBS^a^	32 (69.6)	6 (13.0)	8 (17.4)
JA-OBS Preschool^b^	41 (89.1)	0	5 (10.9)

^a^ JA-OBS Positive outcome = failure on ≥2 of the five items.

JA-OBS Negative outcome = failure on ≤1 of the five items.

^b^ JA-OBS Preschool Positive outcome = failure on ≥2 of the seven items.

JA-OBS Preschool Negative outcome = failure on ≤1 of the seven items.

### 3.3. JA-OBS preschool

All 41 children screened positive on JA-OBS Preschool, i.e., failed on ≥2 of the seven items, and 75.6% failed on ≥6 of the seven items (Table 3).

**Table 3:** Total score on JA-OBS preschool, number (*n*) and percentage (%), *n* = 41.

Number of failures on JA-OBS Preschool	Number of children (%)
1	0
2	2 (4.9)
3	4 (9.8)
4	3 (7.3)
5	1 (2.4)
6	5 (12.2)
7	26 (63.4)

**Figure 2:**
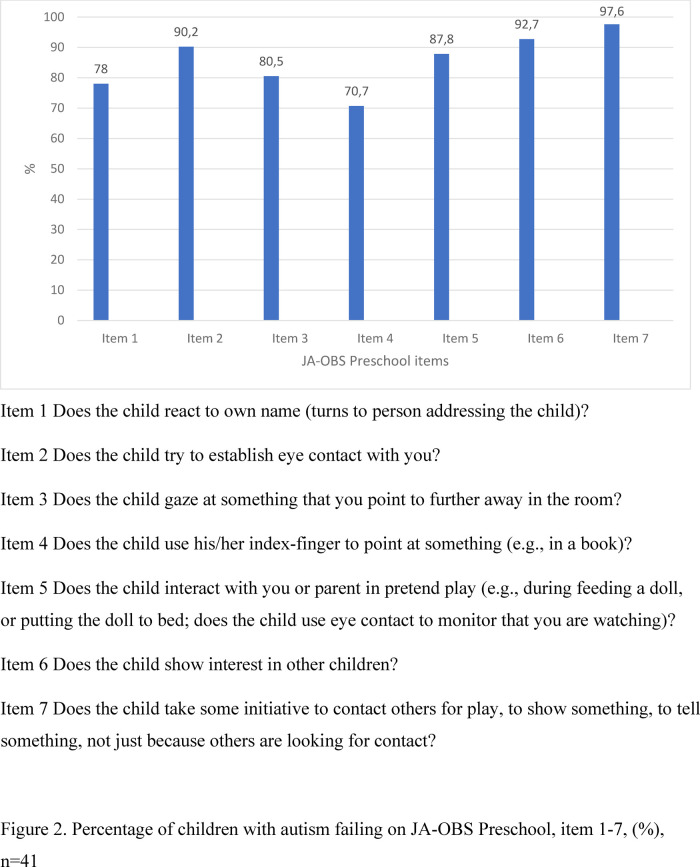
Percentage of children with autism failing on JA-OBS preschool, item 1–7, (%), *n* = 41.

The failure on the respective item on JA-OBS Preschool ranged from 70.7% to 97.6% (Figure 2).

#### 3.3.1. Internal consistency reliability

The internal consistency reliability was studied in 41 observations. Internal reliability was analyzed for all items of the JA-OBS Preschool and calculated at 0.80 (Cronbach's alfa).

#### 3.3.2. Inter-rater consistency

Percent agreement of the JA-OBS Preschool was studied for 13 children, where the results from two examiners who completed the JA-OBS Preschool independently of each other where compared. With both examiners, all 13 children screened positive for autism (failure on ≥2 of the seven items). Percent agreement for individual items ranged from 76.9% to 100% (Table 4).

**Table 4:** Number of failures on respective item for each examiner and percent agreement between two examiners in JA-OBS preschool (*n* = 13).

	Number of failures on respective item for each examiner, *n* (%)		Inter-rater consistency
JA-OBS Preschool	Examiner 1^a^	Examiner 2^b^	Percent agreement
Item 1	10 (76.9)	11 (84.6)	76.9
Item 2	12 (92.3)	13 (100)	92.3
Item 3	12 (92.3)	12 (92.3)	84.6
Item 4	11 (84.6)	10 (76.9)	76.9
Item 5	12 (92.3)	11 (84.6)	76.9
Item 6	13 (100)	12 (92.3)	92.3
Item 7	13 (100)	13 (100)	100

^a^ Examiner 1, the psychologist from the assessment team.

^b^ Examiner 2, special education teacher from preschool.

Item 1 Does the child react to own name (turns to person addressing the child)?.

Item 2 Does the child try to establish eye contact with you?.

Item 3 Does the child gaze at something that you point to further away in the room?.

Item 4 Does the child use his/her index-finger to point at something (e.g., in a book)?.

Item 5 Does the child interact with you or parent in pretend play (e.g., during feeding a doll, or putting the doll to bed; does the child use eye contact to monitor that you are watching)?.

Item 6 Does the child show interest in other children?.

Item 7 Does the child take some initiative to contact others for play, to show something, to tell something, not just because others are looking for contact?.

## 4. Discussion

Although the importance of identifying autism at an early age has been highlighted in many studies, early identification and diagnosis has not yet become the norm (17, 19, 62, 63, 82, 83). Moreover, children from multiethnic and socioeconomically disadvantaged settings are typically diagnosed with autism later than their general peers (8, 44, 66, 68), leading to missed opportunities to provide treatment during possibly critical and sensitive developmental periods (18, 20, 66). Several studies have highlighted the value of preschool staff as partners in identification and referral for children with suspected autism (37, 52, 58, 84–86).

However, introducing detection of signs of autism in preschool requires certain basic prerequisites, such as valid instruments and routines for detection. In the present study, we explored the psychometric properties of the JA-OBS Preschool in children with suspected autism. The JA-OBS Preschool seems to be a promising instrument to detect autism symptoms in this setting. All included children with suspected autism screened positive on the JA-OBS Preschool, and 88% of them had a positive outcome on both JA-OBS and JA-OBS Preschool. The high number of positive screened children on the JA-OBS Preschool indicates high sensitivity. Also, the internal consistency reliability of JA-OBS Preschool was good. In addition, the instrument JA-OBS for use in health care has in an earlier study been found to have good psychometric properties (sensitivity 86% and positive predictive value 92.5%) (Nygren et al., 2012). In the present study, we cannot examine the specificity of the instruments, then all children had suspected autism. Therefore, further studies are needed to determine the psychometric properties of the JA-OBS Preschool in a general child population.

The percent agreement in the rating of JA-OBS Preschool was high and ranged from 77% to 100%, which could indicate an actual agreement between the two examiners. This would also be in line with the results of the JA-OBS inter-rater reliability study (45). Nygren et al. found that an agreement for total scores was obtained in 93% of the observations. Other studies have presented similar results, for example, Nilsson Jobs et al. (58) found that, compared to parents, preschool staff rated autism symptoms more in accordance with the health care staff. In line with a study by Zhang et al. (51), we believe that the special education teachers' special knowledge and experience were crucial for detection of signs of autism and level of agreement between the two examiners. In the present study, the preschool special education teachers were able to recognize developmental impairment in respect of joint attention behaviours. They also had knowledge and experience of working with children with autism and other developmental disorders.

In the JA-OBS Preschool, items 6 and 7 highlight the child's interest in other children and the child's ability to initiate contact and play with other children. Most of the children screened positive on these items (93% respectively 98%), and the percent agreement between the two examiners was high (ranged from 92% to 100%). Items 6 and 7 can be observed in a preschool setting and might complement the observation made in health care. Thus, the structured observation, the JA-OBS Preschool, can also provide valuable information in the continued diagnostic process.

In addition to valid instruments, routines for the detection of autism signs in children are important. At the CHC, the autism screening is general. However, in preschool, we propose using the JA-OBS Preschool when there is any concern about the child's social communication and play development. The observation provides support for the preschool teachers in the judgement and interpretation of autism signs. Routines for detecting autism are essential as they also induce the preschool teachers to pay attention to early signs of autism that otherwise may be unnoticed. The use of the JA-OBS and the JA-OBS Preschool in parallel in different settings can provide additional benefits. It may contribute to the health care and preschool staff receiving a common language to talk about the child's deviant development. Good conditions for the detection of autism signs can be created if a close collaboration occurs between CHC and the preschool.

Opportunities for continuing education and supervision for preschool staff are essential. The education needs to focus on the typical development of communication and interaction, including joint attention behaviours, and early signs of autism, as well as the practical use of the instrument JA-OBS Preschool. Approaching parents about potential concerns regarding the child's development might be challenging for several reasons. Some parents may not be ready to take in that their child might have developmental problems. Also, cultural factors (e.g., differences in the norms of typical child development as well as deviant development, stigma and discrimination, and inadequate understanding of the health care system) might be barriers to detecting early signs of autism (8). It is crucial to provide preschool teachers with support and knowledge to find a sensible way to approach the parents about the child's difficulties. Thus, the instrument needs to be used in a context where there are opportunities for assessment and interventions. The preschool teachers need knowledge about the health care system, so they can guide parents to contact healthcare services for further assessments. We find this to be particularly important for parents with a migrant background because they often have difficulty navigating through the health care system (87).

The first autism signs are often noticeable during the second year of life. In the present study, the average age for the diagnosis of autism was pretty high (38 months). It is desirable to identify symptoms of autism earlier. Therefore, in future study, there is a need to validate the JA-OBS Preschool in a younger age group, children aged 18–24 months.

In Linnsand et al. (7), we proposed a new model to increase accessibility to care, with a local multiprofessional team for both assessment and interventions for young children with autism and their families. Together with autism screening at CHC, we suggest that identification of autism symptoms at preschool may be an essential part of this concept. Information from multiple settings, as well as several informants, may facilitate early identification. Even other studies have indicated that implementation of methods for detection of autism in preschool may be effective and might help improve access to early diagnosis and reduce possible healthcare disparities (56, 57). Thus, further studies should also focus on the use of JA-OBS Preschool and its impact on early diagnosis.

### 4.1. Limitations and strengths

The same multiprofessional team assessed all children in this study, and consistent diagnostic criteria and diagnostic instruments were used.

For a retrospective study like this, the quality of the medical records is essential. In the present study, two pediatricians have met al.l children, and both have very long clinical experience assessing children with different neurodevelopmental and medical disorders, thereby increasing the validity of the data used in this study. In the clinical assessment, to ensure that different areas of development were covered, a medical journal template has been used. Thus, certain data were not available, including missing JA-OBS Preschool for some children.

One limitation of the present study was that the sample consisted of children who had already undergone a previous screening resulting in suspicion of autism. This is likely to have affected the high degree of agreement across examiners. Also, the very high percent of agreement made the use of standard agreement statistics, such as Cohen's Kappa, less appropriate. Instead, we report percentage agreement and highlight that caution should be made in generalizing to other groups on the basis of our results. Further research is needed to determine the psychometric properties of the JA-OBS Preschool in a general child population.The fact that all children had suspected autism and all screened cases were positive, further studies are needed to determine if the observation has the same capacity to confirm the absence of autism signs, i.e., specificity.

Another limitation of the study was the educational level of the preschool special education teacher; the educational level was higher compared to regular preschool teachers. We propose that the high educational level contributes to the high agreement between the special education teacher and the psychologist from the assessment team. Therefore, good knowledge of both the typical development and early signs of autism will be essential in the reliable use of the JA-OBS Preschool.

## Data Availability

The original contributions presented in the study are included in the article/Supplementary Material, further inquiries can be directed to the corresponding author.
